# Potentially inappropriate medications among older adults in Pelotas, Southern Brazil

**DOI:** 10.1590/S1518-8787.2017051006556

**Published:** 2017-06-13

**Authors:** Bárbara Heather Lutz, Vanessa Irribarem Avena Miranda, Andréa Dâmaso Bertoldi

**Affiliations:** I Programa de Pós-Graduação em Epidemiologia. Universidade Federal de Pelotas. Pelotas, RS, Brasil; II Departamento de Medicina Social. Faculdade de Medicina. Universidade Federal de Pelotas. Pelotas, RS, Brasil

**Keywords:** Aged, Self Medication, Drug Therapy, Combination, Drug Utilization, Socioeconomic Factors, Pharmacoepidemiology

## Abstract

**OBJECTIVE:**

To assess the use of potentially inappropriate medications among older adults.

**METHODS:**

This is a population-based cross-sectional study with 1,451 older individuals aged 60 years or more in the city of Pelotas, State of Rio Grande do Sul, Brazil, in 2014. We have investigated the use of medications in the last 15 days. Using the Beers criteria (2012), we have verified the use of potentially inappropriate medications and their relationship with socioeconomic and demographic variables, polypharmacy, self-medication, and burden of disease.

**RESULTS:**

Among the 5,700 medications used, 5,651 could be assessed as to being inappropriate. Of these, 937 were potentially inappropriate for the older adults according to the 2012 Beers criteria (16.6%). Approximately 42.4% of the older adults studied used at least one medication considered as potentially inappropriate. The group of medications for the nervous system accounted for 48.9% of the total of the potentially inappropriate medications. In the adjusted analysis, the variables female, advanced age, white race, low educational level, polypharmacy, self-medication, and burden of disease were associated with the use of potentially inappropriate medications.

**CONCLUSIONS:**

It is important to known the possible consequences of the use of medication among older adults. Special attention should be given to the older adults who use polypharmacy. Specific lists should be created with more appropriate medications for the older population in the National Essential Medicine List.

## RESUMO

**OBJETIVO:**

Avaliar o uso de medicamentos potencialmente inadequados entre idosos.

**MÉTODOS:**

Estudo transversal de base populacional com 1.451 idosos com 60 anos ou mais em Pelotas, RS, em 2014. Investigou-se o uso de medicamentos nos últimos 15 dias. Utilizando os critérios de Beers (2012), verificou-se a potencial inadequação dos medicamentos e sua relação com variáveis socioeconômicas e demográficas, polifarmácia, automedicação e carga de doença.

**RESULTADOS:**

Dentre os 5.700 medicamentos utilizados, 5.651 puderam ser avaliados quanto à inadequação. Destes, 937 eram potencialmente inadequados para idosos segundo os critérios de Beers de 2012 (16,6%). Cerca de 42,4% dos idosos usaram no mínimo um medicamento considerado potencialmente inapropriado. O grupo de medicamentos para o sistema nervoso correspondeu a 48,9% do total de medicamentos potencialmente inadequados. Na análise ajustada, as variáveis sexo feminino, idade avançada, cor da pele branca, baixa escolaridade, polifarmácia, automedicação e carga de doença mostraram-se associadas ao uso de medicamentos potencialmente inadequados.

**CONCLUSÕES:**

É importante que sejam bem conhecidas as possíveis consequências do uso de medicamentos entre idosos. Atenção especial deve ser dada aos idosos que fazem uso de polifarmácia. É necessário existir listas específicas com medicamentos mais adequados para uso em idosos na Relação Nacional de Medicamentos Essenciais.

## INTRODUCTION

The older adults represent a significant population of growing importance in the Brazilian society because of the demographic transition. In this way, new demands arise in terms of public health policies^8^. Chronic conditions have greater visibility in the health care with the aging of the population^15^. In this way, the increased burden of disease and the use of medications become challenges for the system^18^.

Aging is a complex phase and covers many perspectives, such as loss of functions, reduced autonomy, and higher morbidity^18^. Medication is an important instrument for the maintenance and recovery of the health of the older population. The evaluation of pharmacotherapy becomes indispensable in this context. The improvement of the prescription, dispensing, and use of drugs should be a priority in the care programs for the older population^21^.

The older adults are subject to dysfunctions in different organs and, therefore, candidates for polypharmacy (simultaneous use of four medications or more)^20^. A study in Spain has pointed out that the average daily use of medications in this age group is four to eight per person^13^.

Pharmacology in the older adults presents peculiarities because of the decrease in muscle mass and body water. The hepatic metabolism, homeostatic mechanisms, and kidney function may be compromised, which is due to the difficulty in eliminating metabolites, the accumulation of toxic substances, and possible adverse reactions^22^.

The inadequacy of the prescriptions for older patients is a public health problem, given the association with morbidity and mortality, as well as the costs to health services from the adverse reactions^14^.

Potentially inappropriate medications (PIM) are defined as drugs that can provoke side effects that outweigh the benefits in the older adults, in addition to the existence of alternatives to replace them^1^. The PIM continue to be prescribed as first-line treatment for many patients, despite the evidence of poor results in this group^1^.

The PIM obtained considerable recognition after a study conducted in the United States in 1991 by Beers et al. The study reported that 40.0% of residents in nursing homes received at least one PIM^4^. These findings have led to the development of the Beers criteria for PIM for the older population, a list of criteria to guide prescriptions. These criteria were revised and expanded to include all sectors of geriatric care in 1997^5^, 2003^12^, and 2012^1^ and are one of the most used methods^21^. There are few population-based studies in Brazil investigating the prevalence of use of PIM^7,9,23^.

This work had as objective to evaluate the use of PIM in the Brazilian older population.

## METHODS

This is a population-based cross-sectional study with older individuals (aged 60 years or more) in the urban area of the city of Pelotas, State of Rio Grande do Sul, Brazil. This city had 305,696 inhabitants in 2010, of which 46,099 were older persons (IBGE)^a^. The investigation is part of a research consortium of 18 master students of the Graduate Program in Epidemiology (PPGE) of the Universidade Federal de Pelotas (UFPel), entitled “*Avaliação da saúde de idosos da cidade de Pelotas*”, carried out in 2014.

To evaluate the prevalence of use of PIM with margin of error of four percentage points, sample size was estimated at 623 older individuals. To examine the association between the demographic and socioeconomic variables and the use of potentially inappropriate medications, the parameters used in the calculation of sample size were: 95% level of confidence, statistical power of 80.0%, prevalence of outcome of 22.5%^23^, proportions of those exposed and unexposed to the exposures under study according to the literature review and the estimates of the last research consortium of the PPGE/UFPel, prevalence of outcome between the unexposed according to the literature review, and prevalence ratios. In addition, we added 10.0% for losses and refusals, 15.0% for the control of confounding factors, and 1.5 of design effect. The largest estimated sample size was 1,646 subjects to evaluate the association between illiteracy and use of PIM.

The sampling process was carried out in two stages. We selected conglomerates using the 2010 census data^a^. There were 488 sectors in total. As some sectors had a small number of individuals aged 60 years or more, some were grouped resulting in 469 sectors ordered according to the average income for the draw. This allowed the inclusion of several neighborhoods of the city with different economic situations. Each sector contained the information of the total number of households, amounting to 107,152 households in the municipality. Based on the 2010 Census^a^, we included 3,745 households to find the 1,646 individuals. We systematically selected 31 households by sector to enable the identification of at least 12 older individuals, which implied the inclusion of 133 census tracts, which were drawn. The households of the selected sectors were drawn systematically. Persons in the age group studied were invited to participate in the study. We excluded the institutionalized older adults and unable to respond to the questionnaire, who had no caregiver.

Quality control was done by supervisors in 10.0% of individuals in the sample, randomly selected by a reduced questionnaire.

We investigate the use of medications in the 15 days prior to the interview. We asked the respondents to show the packaging or the prescription of the medication used. For each medication, we asked the following question: “Who has indicated this medication for you?”. The answers were: “physician or dentist of the Brazilian Unified Health System (SUS)”, “particular doctor or dentist or those of the health plan”, or “someone else”. The medications were later classified into pharmacological groups using the Anatomical Therapeutic Chemical classification system (ATC)^b^, in the levels 1 (anatomical group) and 2 (therapeutic group).

The demographic and socioeconomic variables used were: gender (male, female), age in three categories (60–69, 70–79, and 80 years or more), self-reported race (white, non-white), economic level classified according to the Brazilian Association of Research Companies (ABEP) (classes A/B, C, and D/E)^c^, education level (zero to three years, four to seven years, eight to 10 years, 11 or more years of study). In addition, we used the variables of polypharmacy (simultaneous use of four drugs or more, according to definition of a recent systematic review^20^), burden of disease, and self-medication.

In the construction of the “burden of disease”, we considered the pathologies: hypertension, diabetes, heart disease, respiratory disease, cerebrovascular accident, arthritis/arthrosis, dyslipidemia, osteoporosis, depression, and cancer. We considered the use of medication as “self-medication” when the older adult reported having consumed at least one medication indicated by a person other than a physician or dentist.

We considered the effect of the sampling design for the analyses, using the set of *svy* commands, specific for the analysis of complex sample-based surveys, of the statistical program Stata 12.1. We performed the description of the sample in relation to the independent variables and calculated the prevalence of the outcome of potentially inappropriate medications with the respective confidence intervals. We calculated the prevalence ratios, 95% confidence intervals, and Wald test for heterogeneity and linear trend (in the case of ordinal variables) in the crude and adjusted analysis.

The update of the Beers criteria published in 2012^1^ was used to analyze potentially inappropriate medications. We questioned the diagnoses that could classify medications as inappropriate, according to these criteria: heart failure, syncope, epilepsy, dementia or cognitive loss, falls or fractures, insomnia, Parkinson’s disease, chronic constipation, gastric or duodenal ulcer, chronic kidney disease, urinary incontinence, and prostatic hyperplasia. We chose to not question the occurrence of delirium, because of the complexity of the diagnosis. In some cases, we also needed to know the dose, dosage, or route of administration of the medication used.

The adjusted analyses were conducted using Poisson regression in two hierarchical levels, keeping in the first level the demographic variables – race, age, and gender – and socioeconomic variables – education and socioeconomic level. In the second level, we included the variables of polypharmacy, self-medication, and burden of disease.

We used the total number of older individuals as denominator for the analysis of the prevalence of potentially inappropriate medications and associated factors. The total number of medications used was used as denominator to characterize the medications for pharmacological groups.

The study was approved by the Research Ethics Committee of the *Faculdade de Medicina da Universidade Federal de Pelotas* (Opinion 472.357/2013). The interviews were conducted after signing the informed consent, with a guarantee of confidentiality.

## RESULTS

The number of older individuals found was 1,844, with 21.3% (n = 393) of losses and refusals, amounting to 1,451 interviews with older individuals. Women accounted for 63.0% of the sample. Most participants were aged between 60 and 69 years, were white, and had up to three full years of study. More than half of the sample belonged to the socioeconomic level C. Approximately 60.0% of the older adults reported up to three morbidities.

Among the respondents, 1,305 reported the use of medication in the 15 days prior to the interview. It was reported the use of 5,700 medications. The most commonly used pharmacological group according to the ATC classification was medications for the cardiovascular system (43.9%), followed by medications for the alimentary tract and metabolism (18.9%), nervous system (15.8%), blood and hematopoietic organs (6.3%), and musculoskeletal system (5.6%). Other pharmacological groups had frequency of use of less than 5.0%.

Among the 5,700 medications, 5,651 were assessed to see if they were inappropriate. Of these, 937 were considered as potentially inappropriate according to the 2012 Beers criteria (16.6%). Among the older adults, 42.4% (95%CI 39.8–44.9) used at least one PIM.

We assessed 5,661 medications regarding the origin of the prescription: 60.3% were prescribed by private physician or dentist or those of health plans and 37.6% were prescribed by physician or dentist of the SUS. Only 2.1% of the medications were used by self-medication. The prevalence of potentially inappropriate medication in private health service or health plan was 15.9% (95%CI 14.7–17.1) and in the prescriptions of the SUS it was 17.1% (95%CI 15.5–18.7).

Among the 898 medications used for the nervous system, 458 were considered as inappropriate. This group accounted for 48.9% of the total PIM. The second group with the highest number of PIM was for the musculoskeletal system. Of that group, 316 medications were used, and 169 were considered as inappropriate (18.0%). Medication for the cardiovascular system corresponded to 15.4% of those classified as potentially inappropriate, and medication for the alimentary tract and metabolism corresponded to 12.5% (Table 1).

**Table 1:** Potentially inappropriate medications for the older adults, according to Beers criteria (2012), according to levels 1 and 2 of the ATC classificationa. Pelotas, State of Rio Grande do Sul, Brazil, 2014.

Levels 1 and 2 of the ATC classification	Potentially inappropriate medications	

	n	%
N – Nervous system (n = 897)	458^b^	48.9^b^
Psycholeptics	217	23.2
Antiepileptics	113	12.1
Psychoanaleptics	105	11.2
Other	23	2.4
M – Musculoskeletal system (n = 316)	169^b^	18.0^b^
Anti-inflammatories and antirheumatics	107	11.4
Muscle relaxants	62	6.6
C – Cardiovascular system (n = 2,481)	144^b^	15.4^b^
Antihypertensives	47	5.0
Cardiac therapy	47	5.0
Calcium channel blockers	34	3.6
Other	16	1.8
A – Alimentary tract and metabolism (n = 1,070)	117^b^	12.5^b^
Medications used in diabetes	96	10.3
Other	21	2,2
Other ATC1 groups (n = 875)	49^b^	5.2^b^

Total	937^c^	100

^a^ ATC: Anatomical Therapeutic Chemical Classification System^26^.

^b^ Values for the categories within the ATC classification.

^c^ We have analyzed 5,651 medications for inappropriate use, of which, 937 were classified as potentially inappropriate according to Beers criteria. The numbers indicated, in parentheses, in each ATC1 group, match what was used by the sample (of the 5,700 medications used by the sample, 61 were missing for some of the information used in the table).

Psycholeptics were the most used PIM, regardless of the dose used or the pathologies presented by the older adults, according to levels 2 and 5 of the ATC, corresponding to 28.6% of the 730 medications in this category, followed by anti-inflammatories and antirheumatics (14.7%), antiepileptics (12.3%), medications used in diabetes (12.2%), muscle relaxants (8.5%), antihypertensives (6.4%), psychoanaleptics (5.2%), and cardiac therapy (3.6%), respectively. The other ATC 2 groups amounted to 8.5% (Table 2).

**Table 2:** Potentially inappropriate medications for the older adults, regardless of dose or diagnosis, according to Beers criteria (2012), according to levels 2 and 5 of the ATC classificationa. Pelotas, State of Rio Grande do Sul, Brazil, 2014.

Levels 2 and 5 of the ATC classification	Potentially inappropriate medications, regardless of dose or diagnosis	

	n	%
N05 – Psycholeptics (n = 231)	209^b^	28.6^b^
Diazepam	48	6.6
Alprazolam	44	6.0
Bromazepam	38	5.2
Other	79	10.8
M01 – Anti-inflammatories and antirheumatics (n = 179)	107^b^	14.7^b^
Diclofenac sodium and potassium	37	5.1
Ibuprofen	13	2.1
Nimesulide	12	1.6
Other	45	5.9
N03 – Antiepileptics (n = 143)	90^b^	12.3^b^
Clonazepam	78	10.7
Other	12	1.6
A10 – Medications used in diabetes (n = 409)	89^b^	12.2^b^
Glibenclamide	88	12.1
Other	1	0.1
M03 – Muscle relaxants (n = 65)	62^b^	8.5^b^
Caffeine + paracetamol + diclofenac sodium + carisoprodol	40	5.5
Orphenadrine citrate + metamizole sodium + caffeine	18	2.5
Other	4	0.5
C02 – Antihypertensives (n = 53)	47^b^	6.4^b^
Doxazosin mesylate	39	5.3
Other	8	1.1
N06 – Psychoanaleptics (n = 329)	38^b^	5.2^b^
Amitriptyline hydrochloride	27	3.7
Other	11	1.5
C01 – Cardiac therapy (n = 172)	26^b^	3.6^b^
Amiodarone hydrochloride	24	3.3
Other	2	0.3
Other pharmacological groups (n = 4,049)	62^b^	8.5^b^

Total	730^c^	100

^a^ ATC: Anatomical Therapeutic Chemical Classification System^26^.

^b^ Values for the categories within the ATC classification.

^c^ Among the 937 potentially inappropriate medications, according to the Beers criteria, 730 are considered potentially inappropriate regardless of dose or diagnosis. The numbers indicated, in parentheses, in each ATC2 group, match what was used by the sample (of the 5,700 medications used by the sample, 70 were missing for some of the information used in the table).

Among the PIM in the older adults regardless of the dose, dosage, or route of administration used, according to the Beers criteria (2012), digoxin showed the highest proportion of inappropriate use because of dose (55.3% of the persons who used the medication had potentially inappropriate dose). Among the women who used estrogens (n = 7), five did not use vaginal preparations, considered as safer. The inappropriate use of acetylsalicylic acid and associations was 3.1% (doses above 300 mg/day are considered as potentially inappropriate). The proportion of inappropriate use was 39.4% (dose above 25 mg/day) for spironolactone and associations. Regarding the use of insulin, 21.9% of the older adults used a variable dose of this medication, which can increase the risk of hypoglycemia (Table 3).

**Table 3:** Potentially inappropriate medications for the older adults, regardless of dose, dosage, or route of administration, according to Beers criteria (2012). Pelotas, State of Rio Grande do Sul, Brazil, 2014.

Active ingredient	N used	Inappropriate	

		n	%
Acetylsalicylic acid and associations	212	6	3.1
Digoxin	42	21	55.3
Spironolactone and associations	38	13	39.4
Insulin	35	7	21.9
Estrogens	7	5	83.3
Doxepin	0	-	
Reserpine	0	-	

Total	334	52	17.0

Missing: 16 ASA + associations, four digoxins, five spironolactones + associations, three insulins, and one estrogen.

Table 4 shows PIM according to diagnoses presented by the older adults. Among these medications, the most used was fluoxetine, with percentage of potentially inappropriate use of 34.7%, because of the history of falls or fractures. Other medications considered as potentially inappropriate depending on a diagnosis were: diltiazem, with inappropriate percentage of 61.0% because of chronic constipation or heart failure (recommended to be avoided in systolic heart failure), citalopram (35.0% of inappropriate use because of falls or fractures), cilostazol (20.5% of inappropriate use because of heart failure), and sertraline (41.0% of inappropriate use because of falls or fractures). Other diagnoses commonly involved in inappropriate medication were cognitive loss, urinary tract symptoms, and prostatic hyperplasia.

**Table 4:** Potentially inappropriate medications for the older adults according to diagnoses, according to Beers criteria (2012). Pelotas, State of Rio Grande do Sul, Brazil, 2014.

Medication*	N used	Inappropriate n	%	Diagnoses involved
Fluoxetine hydrochloride	49	17	34.7	History of falls or fractures
Diltiazem hydrochloride	41	25	61.0	Heart failure; Chronic constipation
Citalopram hydrobromide	40	14	35.0	History of falls or fractures
Cilostazol	39	8	20.5	Heart failure
Sertraline hydrochloride	39	16	41.0	History of falls or fractures
Phenytoin	27	15	55.6	History of falls or fractures
Paroxetine hydrochloride	22	13	59.1	History of falls or fractures
Escitalopram oxalate	18	7	38.9	History of falls or fractures
Verapamil hydrochloride	10	5	50.0	Heart failure; Chronic constipation
Sulpiride	9	8	88.9	Dementia/cognitive loss; History of falls or fractures; Parkinson’s disease; Lower urinary tract symptoms; Benign prostatic hyperplasia; Chronic constipation
Gabapentin	8	2	25.0	History of falls or fractures
Ipratropium bromide	7	2	28.6	Lower urinary tract symptoms; Benign prostatic hyperplasia
Carbamazepine	6	2	33.3	History of falls or fractures
Tramadol hydrochloride	6	1	16.7	Epilepsy
Ranitidine hydrochloride	5	4	80.0	Dementia and cognitive loss
Loratadine	5	4	80.0	Dementia and cognitive loss; Lower urinary tract symptoms; Benign prostatic hyperplasia; Chronic constipation
Oxybutynin hydrochloride	4	2	50.0	Chronic constipation
Isometheptene + dipyrone + caffeine	4	1	25.0	Insomnia
Topiramate	4	1	25.0	History of falls or fractures
Oxcarbazepine	3	1	33.3	History of falls or fractures
Pregabalin	3	1	33.3	History of falls or fractures
Meclizine hydrochloride	2	2	100	Dementia and cognitive loss; Lower urinary tract symptoms; Benign prostatic hyperplasia; Chronic constipation
Desloratadine	2	1	50.0	Dementia and cognitive loss; Lower urinary tract symptoms; Benign prostatic hyperplasia; Chronic constipation
Tiotropium bromide	1	1	100	Lower urinary tract symptoms; Benign prostatic hyperplasia
Cimetidine	1	1	100	Dementia and cognitive loss
Solifenacin succinate	1	1	100	Dementia and cognitive loss; Lower urinary tract symptoms; Benign prostatic hyperplasia; Chronic constipation
Sodium valproate	1	1	100	History of falls or fractures

Total	357	156	43.7	

* Medications considered as inappropriate regardless of diagnosis are not included in this table. Delirium was not assessed in the questionnaire (potentially inappropriate use with this diagnosis: tricyclic antidepressants, anticholinergics, benzodiazepines, chlorpromazine, corticosteroids, H2-receptor antagonists, meperidine, sedative hypnotics, thioridazine).

Table 5 shows the prevalence of the use of PIM and crude and adjusted analyses of this outcome according to demographic and socioeconomic variables, self-medication, burden of disease, and polypharmacy. In the first hierarchical level, all variables of the model, except socioeconomic level, remained associated with outcome after the adjusted analysis: female (PR = 1.26; 95%CI 1.11–1.44), increasing age (PR = 1.25 for 80 years or more; 95%CI 1.07–1.45), protection for non-white race (PR = 0.79; 95%CI 0.68–0.93), low educational level (PR = 1.34 for up to three years of study; 95%CI 1.12–1.60). In the second hierarchical level, three variables were included, all maintaining statistically significant associations with the outcome: polypharmacy (PR = 2.29; 95%CI 1.93–2.70), self-medication (PR = 1.41; 95%CI 1.19–1.67), and burden of disease (PR = 1.64 for four comorbidities or more; 95%CI 1.09–2.45).

**Table 5:** Prevalence of potentially inappropriate medications according to Beers criteria (2012), according to demographic and socioeconomic variables, polypharmacy, self-medication, and burden of disease. Pelotas, State of Rio Grande do Sul, Brazil, 2014. (n = 1,451 older individuals)

Variable^a^	n	Inappropriate (%)	Crude PR	95%CI	p	Adjusted PR	95%CI	p
	Level 1							

Gender					0.001^b^			< 0.001^b^
Male	537	36.7	1			1		
Female	914	45.7	1.25	1.10–1.42		1.26	1.11–1.44	
Age (years)					0.001^c^			0.016^c^
60 to 69	756	39	1			1		
70 to 79	460	45.2	1.16	1.02–1.32		1.11	0.97–1.27	
80 or more	230	48.7	1.25	1.07–1.45		1.18	1.01–1.38	
Race					0.028^b^			0.004^b^
White	1,211	43.7	1			1		
Non-White	236	36.4	0.83	0.71–0.98		0.79	0.68–0.93	
Education (years of study)					0.001^c^			0.001^c^
11 or more	316	36.1	1			1		
8 to 10	143	37.1	1.03	0.81–1.31		1.04	0.82–1.32	
4 to 7	445	41.6	1.15	0.95–1.40		1.17	0.97–1.41	
0 to 3	533	48.2	1.34	1.12–1.60		1.35	1.12–1.62	
Economy level (ABEP)					0.043^c^			0.979^c^
A/B	483	38.5	1			1		
C	720	43.1	1.12	0.97–1.28		1	0.85–1.18	
D/E	169	45.6	1.18	0.98–1.42		0.99	0.80–1.23	

	Level 2							

Polypharmacy					< 0.001^b^			< 0.001^b^
No	746	23.1	1			1		
Yes	705	62.8	2.73	2.36–3.14		2.29	1.93–2.70	
Self-medication					< 0.001^b^			< 0.001^b^
No	1,369	41.1	1			1		
Yes	82	64.6	1.57	1.33–1.87		1.41	1.19–1.67	
Burden of disease					< 0.001^c^			< 0.001^c^
No chronic diseases	109	19.3	1			1		
1	200	22.5	1.17	0.73–1.88		1.04	0.64–1.68	
2	269	33.5	1.74	1.15–2.62		1.34	0.88–2.04	
3	282	42.9	2.23	1.46–3.39		1.44	0.93–2.23	
4 diseases or more	570	57.4	2.98	1.37–3.06		1.64	1.09–2.45	

^a^ The variables are grouped into hierarchical levels according to their entry in the model of adjusted analysis. We have kept in the model, only the variables with p < 0.20, ensuring the control for possible confounding factors for the variables of the same level and upper level.

^b^ Wald test for heterogeneity.

^c^ Wald test for linear trend.

The Beers criteria include a category of medications that should be used with caution by the older adults. Among these medications we can mention selective serotonin reuptake inhibitors (N used = 168), serotonin-norepinephrine reuptake inhibitors (N used = 22), antipsychotics (N used = 39), tricyclic antidepressants (N used = 37), and carbamazepine (N used = 6), which can cause the syndrome of inappropriate antidiuretic hormone secretion. Vasodilators (N used = 31) are present in this category, as they may exacerbate episodes of syncope in individuals with previous history. We found 40 older individuals over 80 years of age using acetylsalicylic acid, even without evidence for use in the prevention of cardiac events in this age group.

## DISCUSSION

Adopting the 2012 Beers criteria, 42.4% of the sample used at least one PIM. This prevalence was higher than in studies in India (16.0%)^25^, Nigeria (25,5%)^10^, and Ireland (28.0%)^6^ and it was similar to a study conducted in New Zealand (42.7%)^16^, using the same criteria. This prevalence is also higher than what has been found in Brazilian studies using the 2003 Beers criteria. One of them, performed in Goiânia, State of Goiás, has found prevalence of 24.6%^23^. In São Paulo, State of São Paulo, a prevalence of 28.0% has been found^7^. A study in Ribeirão Preto, State of São Paulo, using the 2012 update has shown prevalence of 59.2%^3^. The higher prevalence in the studies that have used the 2012 criteria may be justified by the addition of medications to the list. In addition, many studies performed with the 2003 criteria do not evaluate medications that are inappropriate because of the dose used or in the presence of certain pathologies. The difference in prevalence can also be explained from the choice of samples, as some studies have used patients from hospitals or outpatient clinics, in addition to differences in the age of the participants.

Other studies have found higher prevalence of women using PIM^7,11,14^. This may be explained by the fact that women are more likely to seek medical help and talk about health problems. In addition, they tend to live longer than men, living with chronic diseases for longer^3,23^.

Studies show the relationship between low educational level and the use of PIM^2,24^. Educational level is directly associated with socioeconomic level. The SUS needs a more suitable scheme of medication for the older adults^23^. The prescribing habits for SUS users tend to be influenced by the relation of the medication available for free^17^. However, in our study, potentially inappropriate prescription rates were similar among users of the SUS and private services or health plans.

The use of potentially inappropriate medications may be more common among younger older adults^3,11^. This may be explained from the greater care physicians have when prescribing medication for older persons. However, other surveys have found association with increased age^14,24^. It is common the increase in the number of complications arising from old age, the increase of visits to one or more physicians, and the need to use drug combinations in older patients^19^.

Our findings differ from most studies in relation to race, showing discreet protection for non-white persons. In our sample, 84.0% of the older population self-reported as being white.

Our study showed low prevalence of self-medication; however, the association of this variable with the use of PIM remained even after adjusting for the other variables. This indicates the need to raise awareness in the older population so that they can avoid the consumption of non-prescription medication.

The relationship between potentially inappropriate medications and polypharmacy is well established and has been presented in other studies^7,14^. The number of comorbidities (burden of disease), which is associated with the use of multiple medications, is a strong predictor of PIM in our work. Knowing that there are other definitions of polypharmacy (simultaneous use of five or more medications, for example), we also analyzed polypharmacy in this way. However, we found similar results in the crude and adjusted analysis considering four medications or more, or five or more.

By using a hierarchical model of analysis, the socioeconomic and demographic variables (gender, race, age, and education) lose statistical significance with the input of the variables of the second level. The explanation for the association between these variables and the outcome is related to characteristics such as polypharmacy, self-medication, and polymorbidity, which most closely affect the outcome.

The most consumed PIM were the medications for the central nervous system, especially the benzodiazepines, in agreement with the literature^9,23^. The older adults have increased sensitivity to benzodiazepines and decreased metabolism for long-acting agents. In general, benzodiazepines increase the risk of cognitive loss, delirium, falls, fractures, and accidents involving vehicles in the elderly^1^.

Another group that deserves to be highlighted is the medication for the musculoskeletal system, especially anti-inflammatories and muscle relaxants. With increased chronic pain in this age group, these medications are consumed frequently, and many can be purchased without prescription^3^.

Among the medications used in diabetes, there was a high consumption of glibenclamide. This medication increases the risk of hypoglycemia in the older adults, as well as the use of sliding scale insulin^1^. In the group of medications with action in the cardiovascular system, we found high use of amiodarone and doxazosin. The latter, used primarily in benign prostatic hyperplasia, presents a high risk of orthostatic hypotension^1^. Amiodarone is associated with multiple toxicities, including thyroid disease, prolonged QT interval on the electrocardiogram, and pulmonary disease^1^.

We observed a high percentage of older individuals with a history of falls or fractures (28.0%), a situation closely associated with aging. The use of selective serotonin reuptake inhibitors was high in that group. Other medications considered as potentially inappropriate especially in that group of older individuals are anticonvulsants, antipsychotics, benzodiazepines, non-benzodiazepine hypnotics, and tricyclic antidepressants. Such medications can impair motor function, producing ataxia, syncope and, additional falls^1^.

Among the strengths of our study, we could evaluate various pathologies involved in potentially inappropriate prescription. In addition, this is a population-based study, covering older individuals of different scenarios, but it has not included hospitalized or institutionalized older adults.

Our study presents limitations as the Beers criteria have been developed only with medication marketed in the United States. In addition, they do not include other types of inadequacies that are not exclusive to aging (e.g., interactions between drugs and therapeutic duplication)^1^.

Professionals must be aware of the possible consequences of the use of these medications in this age group. A possible explanation for the high prevalence of these prescriptions can be found in the fact that the most prevalent chronic diseases start between the fourth and fifth decade of life. They are managed with medications considered suitable for adults, which are not modified when the individual reaches the age of 60. Special care should be given to the older users of polypharmacy.

It is important to elaborate criteria of national prescription, which include medication available in Brazil. In addition, the National Essential Medicine List (RENAME) needs to contemplate appropriate medications for the older adults, as well as increase their availability for users of the SUS. Another recommendation is the greater emphasis in the curricula of universities on the specifics of the use of medication among the older population, informing future professionals about potentially inappropriate prescriptions for that age group.

## Footnotes

a Instituto Brasileiro de Geografia e Estatística. Censo populacional 2010. Rio de Janeiro; IBGE; 2010 [cited 2015 Jan 28]. Available from: http://censo2010.ibge.gov.br/pt/

b WHO Collaborating Centre for Drug Statistics Methodology. Guidelines for ATC classification and DDD assignment. Oslo; 2000.

c Associação Brasileira de Empresas de Pesquisa. O Novo Critério Padrão de Classificação Econômica Brasil. São Paulo; 2010 [cited 2015 Jan 28]. Available from: http://www.abep.org/criterio-brasil
